# Tick-Borne Encephalitis Virus: Seasonal and Annual Variation of Epidemiological Parameters Related to Nymph-to-Larva Transmission and Exposure of Small Mammals

**DOI:** 10.3390/pathogens9070518

**Published:** 2020-06-27

**Authors:** Laure Bournez, Gerald Umhang, Marie Moinet, Céline Richomme, Jean-Michel Demerson, Christophe Caillot, Elodie Devillers, Jean-Marc Boucher, Yves Hansmann, Franck Boué, Sara Moutailler

**Affiliations:** 1 Nancy Laboratory for Rabies and Wildlife, The French Agency for Food, Environmental and Occupational Health and Safety (ANSES), 54220 Malzéville, France; gerald.umhang@anses.fr (G.U.); marie.moinet@agresearch.co.nz (M.M.); celine.richomme@anses.fr (C.R.); jean-michel.demerson@anses.fr (J.-M.D.); christophe.caillot@anses.fr (C.C.); jean-marc.boucher@anses.fr (J.-M.B.); franck.boue@anses.fr (F.B.); 2 Unité Mixte de Recherche Biologie Moléculaire et Immunologie Parasitaire (UMR BIPAR), ANSES, INRAE, Ecole Nationale Vétérinaire d’Alfort, Université Paris-Est, 94700 Maisons-Alfort, France; elodie.devillers@anses.fr; 3 Infectious Disease Department, University Hospital Strasbourg, 67000 Strasbourg, France; Yves.Hansmann@chru-strasbourg.fr

**Keywords:** tick-borne encephalitis virus, *Ixodes ricinus*, ticks, transmission, small mammals, seroprevalence, density of infected ticks

## Abstract

A greater knowledge of the ecology of the natural foci of tick-borne encephalitis virus (TBEV) is essential to better assess the temporal variations of the risk of tick-borne encephalitis for humans. To describe the seasonal and inter-annual variations of the TBEV-cycle and the epidemiological parameters related to TBEV nymph-to-larva transmission, exposure of small mammals to TBEV, and tick aggregation on small mammals, a longitudinal survey in ticks and small mammals was conducted over a 3-year period in a mountain forest in Alsace, eastern France. TBEV prevalence in questing nymphs was lower in 2013 than in 2012 and 2014, probably because small mammals (*Myodes glareolus* and *Apodemus flavicollis*) were more abundant in 2012, which reduced tick aggregation and co-feeding transmission between ticks. The prevalence of TBEV in questing nymphs was higher in autumn than spring. Despite these variations in prevalence, the density of infected questing nymphs was constant over time, leading to a constant risk for humans. The seroprevalence of small mammals was also constant over time, although the proportion of rodents infested with ticks varied between years and seasons. Our results draw attention to the importance of considering the complex relationship between small mammal densities, tick aggregation on small mammals, density of infected questing nymphs, and prevalence of infected nymphs in order to forecast the risk of TBEV for humans.

## 1. Introduction

Tick-borne encephalitis virus (TBEV) is responsible for the most frequent human viral tick-borne disease in Europe. This flavivirus affects the human central nervous system, causing meningitis or meningoencephalitis with long-term sequelae, with the more severe forms progressing into a loss of consciousness, coma, and even death [1]. In western Europe, only the European subtype (TBEV-Eur), the least virulent subtype, is present [2]. TBEV-Eur is mainly transmitted to a host by a tick bite and is maintained in nature by a cycle involving ticks, mainly *Ixodes ricinus*, and small mammals, especially those belonging to the rodent genera *Apodemus* and *Myodes* [2]. *I. ricinus* requires three blood meals during their lifecycle. The larvae mainly feed on small mammals, while nymphs feed on small mammals, medium-sized mammals, birds, and reptiles, and adults on large animals such as ungulates [3]. Each stage of the tick life cycle takes from several months to around one year to develop to the next, so the entire life cycle is generally completed in two or three years, although this can vary from two to six years depending upon the geographical location [4,5]. 

The virus survived transstadially from one life stage of the tick to the next after the moult (e.g., from larva to nymph), and on rare occasions, can be transmitted vertically from a female tick to its eggs [2]. Ticks can also become infected while feeding on a host during the viraemic phase (systemic transmission). However, the duration of viraemia among small mammals and thus their infectivity to ticks are commonly considered short (two to nine days) [6,7,8,9]. A recent experimental study in bank voles (*Myodes glareolus*) suggests that viraemia might last up to 28 days, and therefore be longer than previously thought, but infectivity to ticks was not tested [10]. Therefore, co-feeding transmission between infected nymphs and uninfected larvae when they feed in close proximity to each other on the same host is thought to be the main transmission mode of TBEV-Eur and the most efficient way to maintain TBEV-Eur in a given area [11,12]. This transmission can even occur through immune hosts, although its efficiency is reduced [13]. Thus, an essential prerequisite for TBEV persistence in a given area is the synchronous activity of larvae and nymphs [12,14].

The generational transfer of TBEV from infected nymphs to uninfected larvae via the host upon which they are feeding via co-feeding transmission, and to a lesser extent via systemic transmission, is the critical life history event that influences the epidemiology and the basic reproductive number *R*_0_ of TBEV [12,15]. *R*_0_ is a critical parameter commonly used in epidemiology measuring the capacity of a pathogen to invade a population of susceptible hosts. For TBEV, *R*_0_ can be defined by the number of infected nymphs produced by one infectious nymph in a fully susceptible tick population. *R*_0_ of TBEV and therefore TBEV-cycle persistence is mainly determined by the ratio of ticks to host, in other words, by the substantial aggregation of ticks on a limited number of hosts [12,15,16]. Ticks follow the “20/80 Rule” [17], whereby 20% of the reservoir hosts feed about 80% of the ticks [3,18,19,20,21]. The propensity of ticks to aggregate depends partly on the tick–host contact rate (activity of questing ticks, host and tick abundance, host community structure). For instance, it has been observed that small mammals are subject to greater tick infestation and higher individual loads of ticks when their population densities are low, given that each individual host had a higher probability of being in contact with ticks and larvae density (the most numerous stage feeding on small mammals) is probably relatively constant over time [21,22,23]. Therefore, the intensity of tick aggregation on a host is a useful parameter to monitor. 

**Figure 1:**
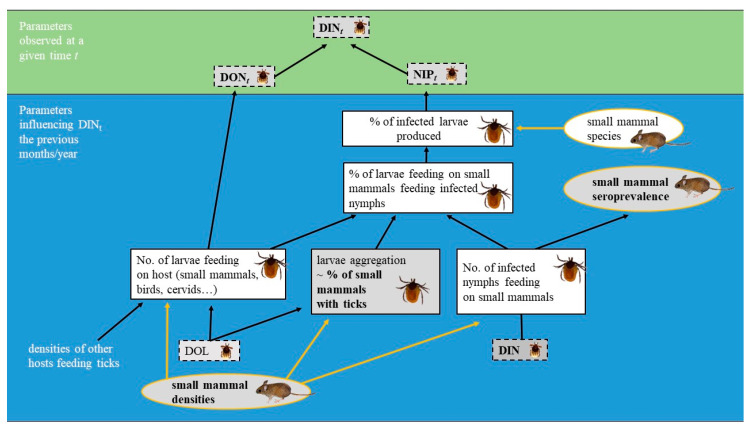
Simplified diagram of the parameters influencing the density of infected questing nymphs at a given time (DIN*_t_*). DON*_t_*: density of questing nymphs at time *t*; NIP*_t_*: prevalence of infected questing nymphs at time *t*; DOL: density of questing larvae. The squares in dotted lines represent the parameters related to ticks questing in the environment, the squares in solid lines represent the parameters related to ticks feeding on small mammals, and the circles in solid lines represent the parameters related to small mammals. The parameters monitored during this study are depicted in bold.

In addition, the density of TBEV-infected nymphs that quest for vertebrate hosts in the environment (DIN for the density of infected nymphs), the prevalence of infected questing nymphs (NIP for the nymphal infection prevalence), and the proportion of small mammals harbouring TBEV-specific antibodies (seroprevalence) are three other relevant ecological and epidemiological parameters to monitor in order to understand the TBEV cycle. As small mammals rarely harbour adult ticks, DIN is an indicator of the instantaneous risk of exposure to TBEV for small mammals and for larvae via the host upon which they are feeding. NIP is a measurement of the proportion of larvae that become infected while feeding on a competent host in the preceding months or year (i.e., the nymph-to-larva transmission rate) and moulted into nymphs. NIP depends on several factors including the intensity of tick aggregation on a few hosts and on the propensity of infected nymphs to feed on small mammals, which can be indirectly measured by the TBEV seroprevalence of small mammals. The relationship between DIN, NIP, and the seroprevalence of small mammals and some parameters that influence these parameters are presented in Figure 1.

The natural foci of TBEV have epidemiological characteristics that vary both intra- and inter-annually. A greater knowledge of these natural foci and their fluctuating characteristics are essential to better assess temporal variations of the epidemiological risks of TBEV-Eur. However, very few studies have addressed this issue [24,25,26]. Both tick (larvae and nymphs) and small mammal densities show large inter-annual and intra-annual fluctuations. The activity of *I. ricinus* depends on humidity and temperature, and is therefore seasonal. Depending upon meteorological and climatic conditions and host availability, the peak abundance for questing larvae is either in late spring–early summer (in northern and central Europe including our study area in eastern France) or in autumn (in western Europe) [4,14,22]. In western and central Europe, the peak for questing nymphs usually occurs in spring and early summer, followed by comparatively low-level activity in mid-summer. In many areas, a second and minor abundance peak is observed in early autumn [4]. In temperate European forests, most rodent species of the genus *Apodemus* and *Myodes* start breeding in spring and their population size reaches a peak in summer or autumn before decreasing during the winter [27,28,29]. Therefore, TBEV nymph-to-larva transmission and exposure of small mammals to TBEV may vary seasonally as tick densities, TBEV-tick prevalence, and their aggregation on hosts varies. Early spring, late spring/early summer and late summer/early autumn are seasons of particular interest for studying the TBEV epidemiological cycle. 

In temperate forests, small mammal populations of the genus *Apodemus* and *Myodes* are also subject to irregular multiannual oscillations, with a year of peak abundance occurring after a year of a heavy seed crop of oak and beech, followed by a year of crashed abundance [27,28,29,30]. By the annual fluctuations in the number of larvae they feed, the temporal variation in the small mammal population may lead to an annual fluctuation in the density of questing nymphs, with a higher nymph density the year after a peak in rodent density [31,32,33]. The effects of these variations on TBEV nymph-to-larva transmission and on the exposure of small mammals to TBEV are not straightforward, as the intensity of tick aggregation also varies annually [21,22]. Another factor that can influence the temporal variation of TBEV nymph-to-larva transmission is the variation over time of the community structure of small mammals (the relative density and the proportion of each small mammal species). Indeed, tick burden and the transmission-competence of the host (i.e., the ability of the host species to facilitate nymph-to-larva transmission through co-feeding or systemic (viraemic) transmission vary from one small mammal species to another such as *Apodemus flavicollis* and *Myodes glareolus* [13,34,35,36]).

France is located on the western border of the known distribution of TBEV, with about ten cases reported each year since the discovery of TBEV in 1968. Most human clinical cases of TBE have been reported in Alsace, a region in the extreme east of France, bordering Germany and Switzerland [37,38]. Contrary to the endemic area of TBEV in these neighbouring countries, the incidence in Alsace is low, with a yearly incidence of 0.5/100,000 inhabitants on average. The epidemiology of TBEV has been poorly studied in Alsace. The only study of TBEV in ticks and small mammals in France was conducted from 1970 to 1974 in a closed peri-urban forest (Neuhof Forest) near Strasbourg, an Alsatian city [25]. We therefore conducted a longitudinal study with a TBEV focus in an Alsatian mountain forest over a 3-year period, from 2012 to 2014. The aims of the present study were to characterise the epidemiology of TBEV in Alsace and to describe both seasonal and inter-annual variations of the TBEV cycle’s epidemiological parameters: (i) the density of TBEV-infected questing nymphs (DIN) and the prevalence of TBEV in questing nymphs (NIP) related to TBEV nymph-to-larva transmission; (ii) the TBEV seroprevalence of small mammals related to their exposure to TBEV; and (iii) the prevalence of tick infestations of small mammals as a proxy for the intensity of aggregation on hosts, since these parameters are well correlated. The seasonal and inter-annual results are then discussed in relation to the variation over time of the density of questing nymphs (DON), the density of small mammals, and the community structure of small mammals. To study the seasonal variation, we defined three seasons: season 1 for early spring (beginning of nymph activity and of small mammal reproduction); season 2 for the end of spring/early summer (peak of nymph activity and of small mammal reproduction); and season 3 for end of summer/early autumn (decrease of nymph and small mammal abundance levels).

## 2. Results

### 2.1. Questing Tick Densities 

**Figure 2:**
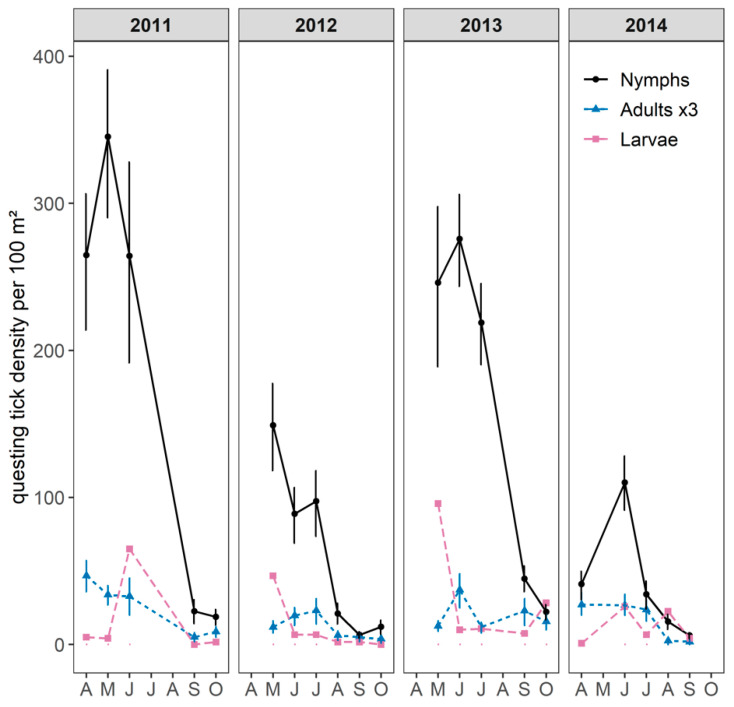
Monthly mean density of questing *Ixodes spp.* larvae, nymphs, and adults per 100 m² from 2011 to 2014 (April to October) with its 95% confidence interval. For the purposes of visual clarity, adult density was multiplied by three and confidence intervals for questing larvae are not shown.

All the ticks collected were identified as the *Ixodes ricinus* species. Overall, questing *I. ricinus* nymphs and adults were found on vegetation in all the sampled months with a unimodal pattern (Figure 2). The main period of activity for both nymphs and adults occurred between April/May and early July. Questing larvae were found from April to October and in higher numbers from May to early July, suggesting a synchronous activity with nymphs. 

**Figure 3:**
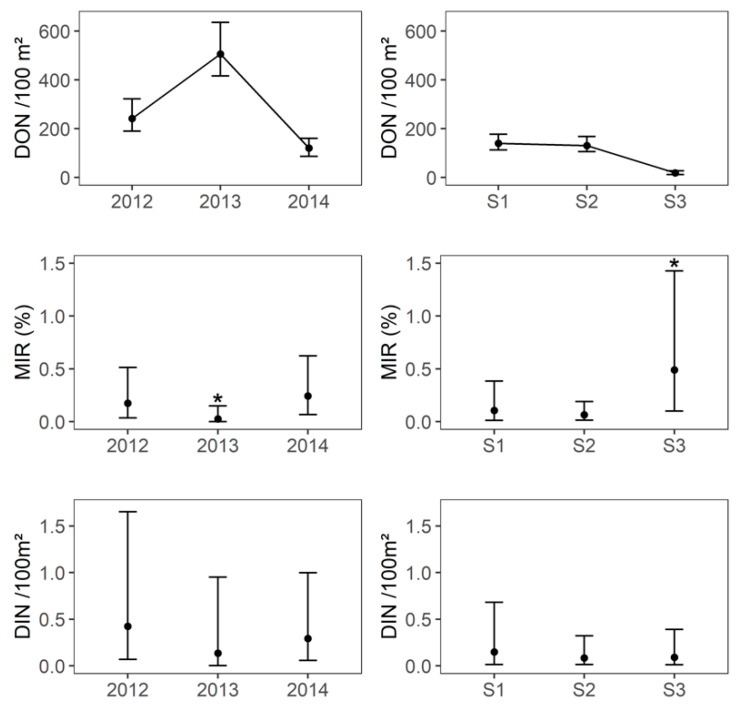
Density of questing nymphs per 100 m² (DON), minimum infection rate of tick-borne encephalitis virus (MIR) in questing nymphs and density of infected questing nymphs (DIN) per 100 m² per year and per season. S1: season 1 (early April–early May), S2: season 2 (early June–early July), S3: season 3 (early September–early October). * The value was significantly different from the others

Questing nymph densities (DON) varied significantly between years (Mann–Whitney *U* test, *p*-value < 0.001). The annual cumulative DON was higher in 2011 and 2013 (500 and 575 nymphs/100 m² with a maximum density per month of 270 and 350 nymphs/100 m², respectively) than in 2012 and 2014 (120 and 240 nymphs/100 m² with a maximum density per month of 110 and 150 nymphs/100 m², respectively), as shown in Figure 2 and Figure 3. The annual cumulative density of questing adults fluctuated in line with the annual cumulative DON, although the annual variations were lower than for the nymphs and varied from 12–18 adults/100 m² in 2012 and 2014 to 25–28 adults/100 m² in 2011 and 2013. 

### 2.2. Tick-Borne Encephalitis Virus Infection Prevalence in Questing Ticks and DIN

A total of 7488 questing *Ixodes ricinus* ticks (7070 nymphs in 570 pools, 219 females and 199 males) were tested for TBEV (Supplementary Table S1). An overall minimum infection rate (MIR) of 0.11% (95% CI: 0.05–0.22) of questing nymphs was observed. MIR in nymphs was significantly lower in 2013 (0.03%, 95% CI: 0–0.15) than in 2012 (0.17%, 95% CI: 0.04–0.51) and 2014 (0.24%, 95% CI: 0.07–0.62, Fisher’s exact test, *p*-value = 0.03), whereas there was no significant difference between 2012 and 2014 (Fisher’s exact test, *p*-value = 0.7, Figure 3). In 2012 and 2014, the MIR in nymphs was significantly lower in seasons 1 and 2 (season 1: 0.13%, 95% CI: 0.003–0.75; season 2: 0.13%, 95% CI: 0.03–0.38;) than in season 3 (0.92%, 95% CI: 0.19–2.67, Fisher’s exact test, *p*-value = 0.02), whereas there was no significant difference between seasons 1 and 2 (Fisher’s exact test, *p*-value = 1, Figure 3). No questing adult was found to be infected (n = 418). The minimum TBEV prevalence that could be detected in adult ticks with a probability of 95%, given the overall sampling size from 2012 to 2014, was 0.7%. This suggests that the overall TBEV infection prevalence in adults was less than 0.7%. 

The annual cumulated DIN appeared lower in 2013 than 2012 and 2014 (Figure 3), but the difference was not significant (2013: 0.14 infected nymphs per 100 m ², 95% CI: 0–0.95; 2012: 0.42 infected nymphs per 100 m², 95% CI: 0.07–1.65; 2014: 0.30 infected nymphs per 100 m², 95% CI: 0.06–1.0). The seasonal DIN did not differ between the three years (Kruskal–Wallis test, *p*-value = 0.9) and between the three seasons (Kruskal–Wallis test, *p*-value = 0.5, Figure 3) and its arithmetic mean per season ranged from 0.09 [95% CI: 0.02–0.32] to 0.15 [95% CI: 0.01–0.68].

The eight sequences obtained from questing ticks (collected in 2012, 2013, and 2014) had 100% identity with each other. One sequence was deposited in GenBank (accession number: MT109187) and showed 99% homology with reference sequences from European subtype strains isolated in ticks (Germany, GenBank KX268728; Finland, GenBank MK801808), and human cerebellum (Finland, GenBank MG589937). 

### 2.3. Small Mammal Abundance 

Over the 15 capture sessions (totalling 45 nights), 1371 captures of small mammals were recorded in total, corresponding to 564 different individuals: 276 *Myodes glareolus*, 287 *Apodemus flavicollis*, and one *Microtus agrestis*. Overall, 342 rodents were captured during two or more trapping sessions. Seven rodents were captured during two different years (five were re-captured in 2013 and two in 2014). We define hereinafter as “rodent-sessions” the number of unique individuals captured per session. The sum of rodent-sessions over the 15 sessions was 906. Data on each individual’s weight and sex were completed for more than 90% of individuals for each trapping session, except in September and October 2012 when 33 individuals died in traps due to the predation by stone marten. 

**Figure 4:**
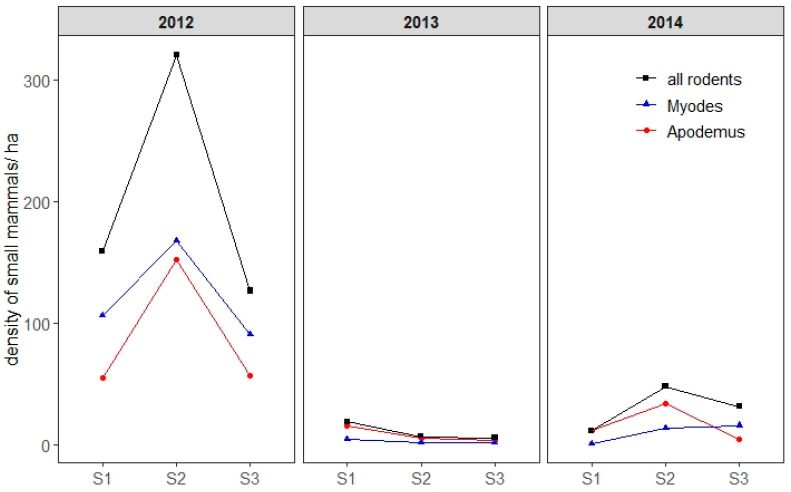
Estimation of the overall density of small mammals for two species in particular, the bank vole (*Myodes glareolus*) and yellow-necked mouse (*Apodemus flavicollis*), per season and per year. Density is represented by the number of small mammals per hectare. S1: season 1 (early April–early May), S2: season 2 (early June–early July), S3: season 3 (early September–early October). For the purpose of visual clarity, confidence intervals are not shown.

The populations of bank voles (*M. glareolus*) and yellow-necked mice (*A. flavicollis*) peaked in 2012 (453 individuals trapped), dropped dramatically in 2013 (32 individuals), and was intermediate in 2014 (85 individuals) (Figure 4). Every year, the density of small mammals peaked during season 2 (June and July). Both species were similarly abundant in 2012 whereas in 2013 and 2014—during the years of the lowest small mammal abundances—*A. flavicollis* was captured slightly more often than *M. glareolus* (Figure 4). In 2012, bank voles were more abundant than yellow-necked mice in April and in September–October. Overall, 74 individuals were captured as juveniles, of which nine (12%), 41 (55%), and 19 (26%) were respectively captured in April, June, and July.

### 2.4. Tick Infestation on Small Mammals

The proportion of rodents infested at least once by ticks significantly varied between years and seasons (Table 1). For all years, the prevalence of tick infestation on rodents was the highest in season 2 compared to the two other seasons (Χ², 2012, *p*-value < 0.001, 2013, *p*-value = 0.001, 2014, *p*-value < 0.001). The prevalence of tick infestation was lower in 2012 than 2013 and 2014 except in season 1. In season 2, this prevalence varied from 44% (95% CI: 38.8–49.3) in 2012 to 94.2% (95% CI: 85.8–98.4) in 2013 and 100% (95% CI: 71.5–100) in 2014. 

**Figure 5:**
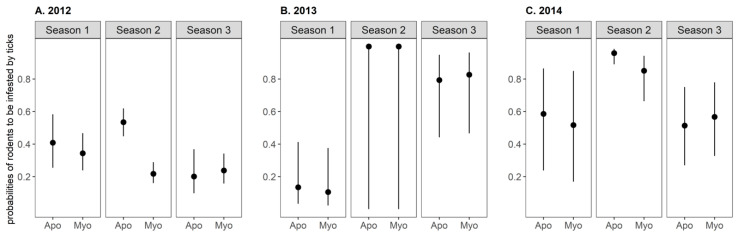
Predicted values of the conditional probabilities of small mammals to be infested by ticks, according to small mammal species and the season for year (**A**) 2012, (**B**) 2013, and (**C**) 2014. These probabilities were calculated for individuals captured once during a season. Values were predicted using a logistic Generalized Linear Model selected according to an Akaike Information Criterion-based model selection procedure (see text for further details). Apo: *Apodemus flavicollis*, Myo: *Myodes glareolus.* Season 1: early April–early May, Season 2: early June–early July, Season 3: early September–early October.

The effects of the small mammal species and sex, season, year, the interaction between season and year, and the interaction between season and small mammal species on tick infestation prevalence were explored following a model selection procedure of logistic generalized linear models (GLMs). The best model included all variables but the sex (Figure 5). Small mammals were significantly more likely to be infested by ticks in season 2 than in seasons 1 and 3. Yellow-necked mice were about twice as more likely to be infested than bank voles in season 2 in 2012. In other seasons and years, there was no significant difference in the prevalence of tick infestation between species. 

A total of 349 larvae of *Ixodes sp*., 17 nymphs of *I. Ricinus*, and four females of *I. trianguliceps* were collected on 152 rodent-sessions in 2012 and 2013. During June and July, the mean number of larvae and nymphs per infested rodent-session (the intensity of tick infestation) was higher in 2013 (5.9 ticks +/− 4.8) than in 2012 (2.2 ticks +/− 2.2) (Mann–Whitney *U* test, *p*-value = 0.01, Table 2) and was not significantly different between species (Mann–Whitney *U* test, 2012: *p*-value = 0.64, 2013: *p*-value = 0.45, Table 2). The proportion of rodent-sessions infested by nymphs and co-infested by larvae and nymphs was higher in 2013 (6/12 and 5/12, respectively) than in 2012 (3/67 and 2/67, respectively, Table 2, Fisher’s exact test, *p*-value < 0.001). As a consequence, only 2.7% (4/127) of larvae fed simultaneously with nymphs in 2012 vs. 57.8% (37/64) in 2013. 

### 2.5. Detection of TBEV Antibodies in Small Rodents

We analysed 448 serum samples from 249 *M. glareolus* and 381 from 274 *A. flavicollis*. Overall, 4.2% [95% CI: 2.6–6.3] of individuals and 2.8% [95% CI: 1.8–4.1] of rodent-sessions were seropositive for TBEV. This proportion did not differ significantly between years (Fisher’s exact test, *p*-value = 0.43), varying from 1.3 % [95% CI: 0.0–7.1] in 2014 (n = 76 individuals) to 4.7% [95% CI: 2.9–7.2] in 2012 (n = 422 individuals). Seroprevalence levels were similar between seasons in 2012 (3.2–5.2%, Fisher’s exact test, *p*-value = 0.53) (Table 1), with TBEV antibodies detected from April to October. In 2013 and 2014, TBEV antibodies were inconsistently detected throughout the year (Table 1). 

Among the 22 seropositive rodents, three and one rodents were re-captured, respectively, one and two months after the detection of their seroconversion. TBEV antibodies were detected in two consecutive months for only one individual. Two juvenile yellow-necked mice (out of 61 juveniles of both species tested) with a body mass of 11 g and 15 g tested seropositive. 

None of the variables included in the logistic model—species, year, and season—had a significant effect on the probability that a rodent would be seropositive. 

### 2.6. Detection of TBEV in Feeding Ticks 

All ticks collected on rodents in 2012 and 2013 were tested for TBEV. In 2012, a MIR of 1.1% [95% CI: 0.2–3.1] was found on larvae (n = 283) and no nymphs (n = 9) or females (n = 4) were found to be infected. No larvae were found positive in season 1. The MIR in larvae did not differ significantly between seasons 2 and 3 (season 2: 0.7% [95% CI: 0.0–3.7], season 3: 4.2% [95% CI: 0.5–14.2], Fisher’s exact test, *p*-value = 0.15). TBEV was detected in larvae feeding on three rodent-sessions (2.2% of rodent-sessions infested by ticks, n = 137): one seronegative *A. flavicollis* captured in June (1/67 rodents infested by ticks during the June session) and two seronegative *M. glareolus* captured in September (2/22 rodents infested by ticks during the September session). These animals were not re-captured later. None of the feeding ticks collected in 2013 were found to be infected. Overall, 31 larvae and one nymph feeding on eight TBEV-seropositive rodents were negative for TBEV. 

The three sequences obtained from feeding ticks (collected in 2012) had 100% identity with each other. One sequence was deposited in GenBank (accession number: MT109186) and showed 100% homology with sequences obtained from questing ticks during our study, and 99% homology with reference sequences from European subtype strains isolated in ticks (Germany, GenBank KX268728; Finland, GenBank MK801808), and human cerebellum (Finland, GenBank MG589937).

## 3. Discussion

This study improves knowledge of the seasonal and inter-annual variation of TBEV nymph-to-larva transmission and small mammal exposure to TBEV. It adds to the only study conducted on the subject in France before this one [25]. Our results confirm a low circulation of TBEV in ticks and rodents on the study site. 

### 3.1. Low Circulation of TBEV in the Studied Site

TBEV was detected in nymphal ticks from 2012 to 2014 with a very low prevalence varying annually from 0.03% [95% CI: 0–0.15]) to 0.24% [95% CI: 0.07–0.62]. Although adult ticks are generally more frequently infected than nymphs [25,39,40,41], our study did not detect the virus in adult ticks. This could nonetheless be explained by our small sample size (n = 418). The method of collecting ticks by blanket dragging is more suitable for collecting nymph ticks than for adult ticks of *I. ricinus*. Considering the number of adults tested for TBEV, its prevalence in adult ticks was estimated to be lower than 0.7%. These estimates fit within the lower range values found in other studies in Europe, ranging from <0.1 to 5% [2], and are similar to those observed by Perez-Eid et al. [25] in the Neuhof forest in Alsace between 1970 to 1974 with a maximum prevalence of 0.12% [95% CI: 0.04–0.44] in nymphs and 0.77% [95% CI: 0.37–1.42] in adults. 

In rodents, anti-TBEV antibodies were detected from 2012 to 2014 in 0%–5.2% of individuals per season. The persistence of TBEV-Eur specific antibodies in rodents is poorly understood. Depending on different studies, TBEV antibodies can be detected between the fifth up to the forty-second day post-infection [42,43] (cited by Perez-Eid et al. [25]) or up to 100–168 days post-infection [44,45]. In our study, the observed disappearance of antibodies in seropositive animals captured a second time and the finding of seronegative animals carrying infected larvae prone to a short half-life of the anti-TBEV antibodies. The latter group of animals may have been recently infected and not yet seroconverted. Therefore, TBEV seroprevalence in small mammals is a good proxy for the occurrence of new infections in a month, although it is impossible to distinguish antibodies due to maternal transfer from those developed after a recent infection. In small mammals, maternal antibodies generally last from six to 10 weeks [46,47,48], but this duration is unknown for TBEV. We captured two juveniles aged 3–4 weeks according to their weight that were seropositive. Considering the time needed for seroconversion after an infection and the probable duration of maternal antibodies, these antibodies were probably maternal antibodies. Although serological tests and sampling design vary according to studies, the seroprevalence we observed in 2012–2015 is similar to that found by Perez-Eid et al. in Alsace [25] (2.4%) and also fits within the lower values found in western and central Europe (prevalence ranging from 1.6% to 23% [44,49,50,51,52,53,54]). In 2012 and 2013, we also observed a small proportion of rodents from which feeding ticks acquired the virus (2.2%) associated with a low prevalence in feeding ticks (1.1%). Similar to other tick-borne pathogens [55], the probability of a feeding tick acquiring the virus might depend on the duration of attachment of the ticks on the hosts. If this is the case, the prevalence in feeding ticks might have been underestimated since the ticks were collected before they finished their meal on the hosts. 

Our results in rodents and questing ticks therefore suggest a very low circulation of TBEV in our site. This strengthens the hypothesis that the virus circulates at a very low level in the natural foci of the Alsace region, located at the western boundary of TBEV distribution, where the low annual human incidence is of 0.5/100,000 inhabitants. From 2012 to 2014, only three to six human cases were reported in Alsace (Hansmann, pers. comm). The strength of TBEV enzootic cycles is influenced by the number of larvae co-feeding with nymphs on small mammals [16,20,56]. Like the results of Perez-Eid et al. [57] in Alsace in the 1970s, we observed a very low number of co-feeding ticks in June–July during the period of the highest tick activity, especially in 2012. We only captured 4.5% of rodents infested by nymphs in 2012 when rodent abundance was high, whereas 17% to 33% of rodents were reported to be infested by nymphs in TBEV-infected sites in Switzerland and Slovakia [18,20]. In addition, the mean infestation of larvae per rodent-session infested by ticks (2.2 larvae per rodent) and per rodent-session infested by nymphs (two larvae per rodent infested by nymphs) in 2012 was also lower than that observed in other TBEV-infected sites (from 10 to 80 larvae per infested rodent and from five to 65 larvae per host infested by nymphs) [18,20,21]. However, the number of feeding ticks observed in our study might be underestimated compared to other studies, since our method for counting feeding ticks (count of ticks on live animals) is not directly comparable with the methods used in other field studies (count of ticks that have dropped off from freshly dead animals or from animals brought in the laboratory). 

### 3.2. Seasonal and Inter-Annual Variation of TBEV Prevalence in Ticks and Rodents in 2012–2014

Although the variations were low, we detected an annual and seasonal effect on NIP (the TBEV prevalence of questing nymphs), but not on DIN or rodent seroprevalence. Our study site in 2012–2014 was characterised by a high inter-annual and inter-seasonal variation in small mammal and questing nymph densities. Annually, the peak of activity for questing larvae and nymphs, of larvae feeding on small mammals, and of small mammal density occurred during the same season (i.e., in June–July). The density of bank voles and yellow-necked mice started to decrease from September. This decrease was greater for yellow-necked mice than for bank voles, only 13% of yellow-necked mice being captured in September–October. This seasonality is similar to field observations in TBEV-infected sites in neighbouring countries [18,20,21], but differs from findings in Brittany, western France, where the peak of small mammal densities and of feeding larvae were found to occur in autumn [22]. Our observations on inter-annual variations comply with previous observations [21,22,23,32,33]: we found that the density of questing nymphs was higher the year following a year of high rodent density and that the intensity of tick aggregation on small mammals was higher when the density of small mammals was low. Indeed, the prevalence of infestation by both stages—larvae and nymphs—and the mean infestation was higher in 2013 (a low-density year for small mammals) than in 2012 (a high-density for small mammals).

NIP was higher in both 2012 and 2014 compared than 2013; this is contrary to the density of questing nymphs, for which we found opposite variations. However, DIN was near-constant over those years. Therefore, our results suggest that the proportion of infected larvae produced was higher in 2013 when more larvae and nymphs fed on the same very few hosts because there were fewer hosts available. This led to a higher proportion of infected questing nymphs the year after. This is consistent with the fact that the proportion of larvae feeding on rodents infested by nymphs was higher in 2013 than 2012. Therefore, the inter-annual difference in NIP can be explained by the variation in aggregation intensity of ticks on rodents along with rodent abundance. However, the density of questing nymphs was lower in 2014 than in 2013. The low density of rodent population in 2013 might have contributed to reduce the overall number of fed larvae, since the densities of the other main hosts for ticks (e.g. cervids and birds) generally display small variations over a 3-year period [31,32,33]. Consequently, the DIN stayed constant over the years. Our study was only conducted over a 3-year period and the results cannot be generalised. Further investigations over a longer period would be needed to better understand the relationship between DON, DIN, NIP, and small mammal density for TBEV. Few studies have investigated these relationships for other tick-borne pathogens amplified by rodents. In North America, Ostfeld et al. [32] studied the relationship between the *Ixodes scapularis* tick, the *Borrelia burgdorferi* sensu lato (Bbsl) bacterium and the white-footed mouse (*Peromyscus leucopus*) for 19 years. They found no effect of white-footed mouse density on NIP_Bbsl_ whereas DIN_Bbsl_ was positively, but not linearly, related to mouse density in the previous year. At low mouse density values, DIN _Bbsl_ was almost constant. In a short-term study in the Netherlands, Krawczyk et al. [33] observed that the relationship between NIP, DIN, and the rodent density the previous year depended on the transmission mode of the tick-borne pathogens. Contrary to most tick-borne pathogens amplified by rodents, TBEV is mainly transmitted by co-feeding and the nymph-to-larva transmission of TBEV strongly depends on the intensity of tick aggregation on small mammals. These elements probably induced a different relationship between DIN, NIP, and rodent density that should be investigated further. 

Although the difference was small, TBEV prevalence in questing ticks was higher in autumn than in spring. This finding had already been observed in the previous study conducted in Alsace [25] and in studies targeting other tick-and-rodent-borne pathogens in Germany and Luxembourg [58,59]. There are no data on the timing of diapause and moulting of *I. ricinus* stages in the Alsace region. However, if larvae that feed early in the season (in April–May) become nymphs that quest later the same year (in July–October), as observed in Switzerland [5], then this could be a potential mechanism to explain the higher prevalence in questing nymphs in autumn. Indeed, TBEV transmission to larvae might be higher in June–early July since this period coincides in our study area with the peak abundance of questing larvae and nymphs, of larvae feeding on small mammals, and of small mammals themselves, especially *Apodemus sp*., which has been shown experimentally to be more efficient in fostering TBEV transmission than *M. glareolus* [45,60]. Moreover, the virus titre in the nymphal tick may drop over time and in unfed ticks undergoing a winter diapause, as observed experimentally (Mishaeva and Erofeeva [61] cited by Perez-Eid et al. [57]), which would also induce lower prevalence in early spring. 

Surprisingly, from 2012 to 2014, we did not detect any annual or seasonal effects on the TBEV seroprevalence in rodents whereas the prevalence of tick infestation on rodent and tick load per rodent varied and DIN values were constant. A higher seroprevalence could be expected in those years or seasons of low rodent abundance, along with the higher prevalence of tick (nymph) infestation on rodents and nymph load per rodent, as observed in other studies [50] and for other tick-borne pathogens amplified by rodents [22]. Similarly, there was no species effect on the TBEV seroprevalence despite *A. flavicollis* being found to be more infested by ticks in this study (especially in spring when *A. flavicollis* is abundant). This apparent contrast may result from a difference in the immune response between species with a lower TBEV antibody titre and persistence in *A. flavicollis* compared with *M. glareolus* [7,45,51,62,63]. We could then have expected to see an annual or seasonal difference in the infection probability per species, but our sample size of rodents was probably too small to detect a significant difference given the low seroprevalence of rodents, the low persistence of the antibodies, and the low DIN. Another explanation for the absence of any annual effect on small mammal seroprevalence could be a lower detection of seropositive small mammals the years when they were scarce. Indeed, during those years, small mammals may acquire the infection much earlier in the year given the high tick aggregation level when fewer hosts are available. The exposure to this infection would probably remain undetected since the half-life of the anti-TBEV antibodies seems short in small mammals. 

In conclusion, this study shows that the virus was circulating at a very low level in our study site. Despite this very low-level circulation, we were able to observe significant variation in the inter-annual and inter-seasonal prevalence of TBEV in questing nymphs, indicating that the nymph-to-larva transmission of TBEV varied over time. However, the density of questing TBEV-infected nymphs showed no detectable variations over time, suggesting that the rate of exposure for humans was probably relatively constant over the period of the study. The seroprevalence of small mammals was constant over time although the prevalence of tick infestation varied on an annual and seasonal basis. More studies are needed to understand the relationship between the density of TBEV-infected questing infected nymphs, the density of questing nymphs, the prevalence of TBEV in questing nymphs, TBEV seroprevalence in small mammals, and small mammal density.

## 4. Materials and Methods

### 4.1. Study Area

The study focussed on a 4.0-hectare area at Murbach (47°55’03N, 07°08’46E; average altitude of 630 m) in Guebwiller Valley, Alsace, which is the region where most of the human cases of TBEV have been reported in the last two decades [37] and where the virus was isolated from questing ticks in 2010 (unpublished data). The site is covered by mixed forests classified as *Asperulo-Fagetum* beech forests, with a predominance of *Fagus sylvatica* beech and *Abies alba* European silver fir. 

### 4.2. Questing Tick Sampling

Questing ticks were counted and collected by dragging a 1-m² white blanket over the same area as the rodent trapping grid (see below). The trapping grid was divided into 16 quadrats and the blanket was dragged over three different 10-metre-long transects within each of these 16 quadrats. In all, 48 different transects totalling a surface area of 480 m² were investigated within the 4.0-hectare area. Ticks were collected during the first week of the month, once a month from April to October in 2011, and from May to October in 2012, 2013, and 2014, with the exception of July and August 2011, August 2013, and October 2014, when no ticks were sampled. At the laboratory, ticks were first identified to species level based on their morphology using appropriate keys and descriptions [64]. All questing nymphs and adults were washed in 70% ethanol, rinsed twice in distilled water, dried, and stored at −80°C until tested for TBEV.

### 4.3. Small Mammal Trapping and Sampling

Small mammals were trapped from 2012 to 2014 five times per year, in mid-April and in each first week of June, July, September, and October. The trapping grid consisted of 196 live-traps (14 × 14 Uggland special no. 3, Grahnab, Sweden) set at 15 m intervals, covering a total area of 4.0 ha. For each session, traps were set for three consecutive nights and baited with carrots and sunflower seeds. Trapped rodents were individually marked with a transponder (Vétérissimo Mini RWI-I, Vethica, France). Blood samples were taken once per session through the retro-orbital sinus. Species, body mass, sex, and tick presence were recorded and then the animals were released at the point of capture. The whole body of the animals were inspected to detect tick presence, with more attention given to the head, ears, and neck. Given that the sexual maturity in rodents is reached around 6–8 weeks [65], we used the minimum value of weight of all individuals for each species observed during a second capture session (i.e., aged of at least 6–7 weeks) to define the threshold value below which an individual was considered juvenile. Accordingly, individuals weighing less than 14 g for *M. glareolus* and 16 g for *A. flavicollis* were considered juveniles. From April 2012 to July 2013, all the ticks found on rodents were collected. In June and July 2012, given the high number of small rodents captured, although all the animals were subject to blood sampling and examined for tick presence, we only counted and collected the ticks of a number of randomly-chosen animals infested by ticks (one every three rodents infested by ticks). All the ticks and blood samples were kept at +4°C until brought back to the laboratory. There, the blood clot was gently detached from the bottom of the Eppendorf tube before being centrifuged at 5000 rpm for 5 min and the serum obtained was stored at −20 °C until tested for the presence of TBEV antibodies. Larvae were identified at genus level and nymphs/adults at species level based on their morphology using appropriate keys and descriptions [64]. All the ticks were washed in 70% ethanol, rinsed twice in distilled water, dried, and stored at −80 °C until tested for TBEV. 

### 4.4. Ethical Statement 

The experimental protocol with small mammals complied with EU Directive 2010/63/EU and was submitted to and approved by the French Ministry of Research (APAFIS No. 2015120215112678). All efforts were made to minimize animal suffering. The species studied are not protected in France or included in the International Union for Conservation Nature Red List of threatened species in France. The animal trapping took place with permission from the landowners. 

### 4.5. Laboratory Analysis 

#### 4.5.1. TBEV Antibody Detection in Rodents

Serum samples were screened for the presence of TBEV antibodies, using an in-house indirect enzyme-linked-immuno-sorbent-assay (ELISA) using a domain III TBEV envelop as an antigen developed to capture specific IgG antibodies. ELISA plates were coated (2 µg/well) with a purified SNAP-TBEV EDIII recombinant protein or a non-relevant SNAP (soluble NSF attachment protein). The non-relevant SNAP is the SNAP label expressed from an empty expression vector. The recombinant SNAP-TBEV EDIII protein and non-relevant SNAP were produced as described in Beck et al. [66]. After overnight coating with antigen, the plates were saturated with 1% Bovine serum albumin (BSA) in phosphate buffered saline (BPS) solution. Assays were performed in duplicate with 100 µL of serum samples (diluted 1:500 in PBS), tested on SNAP-TBEV EDIII recombinant protein and on the SNAP production on the same plate. Following incubation for 1 hour at 37 °C, the plates were washed three times in PBS-Tween 0.05%. The second antibody was a polyclonal goat anti-mouse conjugated with horseradish peroxidase (Dako), diluted to 1:500. After 1 hour at 37 °C, the wells were washed four times with 300 μL of washing buffer. The bound peroxidase was revealed with 100 µL 1.8 mM of o-Phenylenediamine (Invitrogen) in PBS and 0.02% H_2_O_2_, according to the manufacturer’s instructions. After 40 min in the dark at room temperature, the enzyme reaction was stopped with 0.5 M H_2_SO_4_, and the optical density (OD) was measured at 492 nm on an automatic plate reader.

The positive controls used to validate the ELISA plate protocol consisted of serum from two groups of three Swiss mice that had previously been immunised twice with the recombinant SNAP-TBEV EDIII protein and used at a dilution of 1:5000. The negative controls were three Swiss mice that had not been immunised. The samples were considered positive when the duplicate mean OD values read for SNAP-TBEV EDIII minus the mean OD values read for SNAP was higher than 0.100 OD.

#### 4.5.2. TBEV Detection in Questing and Fed Ticks 

Only ticks collected from 2012 to 2014 were analysed. For questing ticks, adults were analysed individually and nymphs were analysed in pools of one to five ticks. For fed ticks (collected on small mammals), larvae/nymphs were pooled per stage, engorgement status (fed, unfed), animals, and month of capture with one to ten ticks per pool, and adults were analysed individually. Ticks were homogenised using 2.8 mm stainless steel beads in a Precellys 24 lyser/homogeniser (Bertin, France) at 5500 rpm for 20 s. RNA was extracted using the Nucleospin RNA II kit (Macherey Nagel, Düren, Germany), according to the manufacturer’s instructions. Purified RNA was eluted into 50 µL RNase-free water and stored at −80 °C until use. RNA samples were screened for TBEV by real-time RT-PCR (reverse transcription-polymerase chain reaction) targeting a 3′ non-coding region of the TBEV genome with specific primers and probes [67]. Real-time RT-PCR Taqman assays were performed in a final volume of 20 µL using the LightCycler 480 RNA Master Hydrolysis Probe master mix (Roche Applied Science, Penzberg, Germany), according to the manufacturer’s instructions using 2 µL of RNA template. Positive and negative (water) controls were included in each run. Real-time RT-PCR thermal cycling conditions were as follows: 63 °C for 3 min, 95 °C for 30 s, 45 cycles at 95 °C for 10 s, then 60 °C for 30 s, followed by cooling at 40 °C for 10 s. Conventional RT-PCR followed by nested PCR using primers targeting the non-structural protein gene NS5 [68] were used to confirm the presence of TBEV in positive samples. Amplicons were sequenced by Eurofins MWG Operon (Germany), and then assembled using BioEdit software (Ibis Biosciences, Carlsbad). The online BLAST tool (National Centre for Biotechnology Information) was used to compare results with the published sequences listed in the GenBank sequence databases. 

### 4.6. Descriptive and Statistical Analyses

#### 4.6.1. Definition of Three Seasons

We defined three seasons: season 1 for early spring (beginning of nymph activity and of small mammal reproduction); season 2 for the end of spring/early summer (peak of nymph activity and of small mammal reproduction); and season 3 for end of summer/early autumn (decrease of nymph and small mammal abundance levels). Given the small number of positives, we grouped data per season. 

For small mammals, season 1 corresponded to the captures of April; season 2 grouped the captures of early June and July; and season 3 grouped the captures of early September and October. For each season, we listed each individual rodent captured along with its tick infestation status, TBEV seropositive status, and the number of captures (one or two). An individual was considered to be infested by ticks in season 2 (respectively season 3) if it was found to be infested by at least one tick in June or/and July (respectively in September or/and October). Similarly, an individual found to be infested by ticks in at least one of the five trapping sessions of the year (from April to October) was considered infested by tick in the year. An individual was considered to be TBEV-seropositive in season 2 (respectively season 3) if it was found to be TBEV-seropositive in June or/and July (respectively in September or/and October). An individual found to be TBEV-seropositive in at least one of the five trapping sessions of the year (from April to October) was considered TBEV-seropositive in the year.

For questing ticks, season 1 corresponded to data for early May; season 2 grouped data for early June and July; and season 3 grouped data for early August, September, and October. 

#### 4.6.2. Descriptive and Statistical Analyses of Tick Density and TBEV Infection Prevalence in Ticks

The monthly density of questing ticks was estimated for each tick stage by the mean density of questing ticks along each sampling transect, and was expressed as the mean number of ticks per 100 m². Its 95% confidence interval (95% CI) was calculated by bootstrapping by measuring the quantiles 2.5% and 97.5% of the mean density over a sub-sampling of 30 transects (among the 48 realized) generated 1000 times. The density of questing ticks was estimated per season by the mean of the density of questing ticks in the corresponding months and its bootstrapped 95% CI was calculated as previously described. The density of questing ticks was estimated per year by summing the tick density for each season (=annual cumulated DON) and its 95% CI was calculated by summing the lower and upper limits of the seasonal tick density. Tick questing density per year was compared using the observed yearly peak density and the annual cumulated DON with a Mann–Whitney U test. 

As TBEV infection prevalence in ticks is usually lower than 1% in Alsace [25,69], prevalence in ticks was expressed as the minimum infection rate per 100 tested (MIR), based on the assumption that a single tick was positive within a positive pool. Exact 95% CIs were calculated on the basis of binomial distribution. For the 2012−2014 period, MIR was calculated per season for each year. The inter-annual and inter-seasonal MIR of questing ticks was compared using Fisher’s exact test. If no TBEV was detected in a sample of ticks, we calculated the minimum prevalence of TBEV that could be detected in ticks with a probability of 95% given the sample size used by applying the formula proposed by Cannon [70]. 

We calculated the density of infected nymphs (DIN) per season by multiplying the seasonal MIR divided by 100 by the seasonal density of questing nymphs, and the DIN per year by multiplying the annual MIR divided by 100 with the annual cumulated DON. We used the lower and upper limits of the 95% CI for MIR and the lower and upper limits of DON per season and year to estimate the lower and upper bounds of the 95% CI for DIN. The seasonal DIN was compared between years and seasons using a Kruskal–Wallis test.

#### 4.6.3. Descriptive and Statistical Analyses of Rodent Density, Tick Infestation, and TBEV Seroprevalence in Rodents

Rodent density was calculated per session and per species according to the standardised closed population Schnabel method that takes into account multiple marking occasions [71]. Since the captures were carried out on three consecutive days per session, we considered that the population was closed for each session. Rodent density per season was calculated using the mean of rodent density estimated for the corresponding months. 

The tick infestation prevalence for rodents was defined as the number of rodents carrying at least one tick divided by the number of rodents inspected. We calculated tick infestation prevalence per year, season, species, and age class (juveniles vs. adults). Exact 95% CIs were calculated using the binomial distribution. We described the infestation of rodents by larvae and nymphs in season 2 (the period of highest nymph density) of 2012 and 2013 by calculating (1) the mean number of larvae and nymphs per individual infested by ticks; (2) the proportion of rodents infested by ticks carrying nymphs only or nymphs and larvae; and (3) the proportion of larvae feeding on rodents that were also carrying nymphs. The results for 2012 and 2013 were compared using a Mann–Whitney *U* test.

The tick infestation status of rodents was modelled using a logistic GLM (generalised linear model) with a binomial distribution and logit link, and a binary response variable (absence of ticks = 0; presence of ticks = 1). The variables considered were the rodent species, sex, season, year, and the interactions between year and season (to allow seasonal patterns of the probability of an individual to have ticks to vary between years) and between season and species (to allow the species effect on the probability of an individual to have ticks to vary according to the season to mimic seasonal variation of rodent community structure and density). We included the number of captures within the season as an offset term. Some animals were captured in several different seasons and constituted temporal pseudo-replication of individual hosts. We therefore ran logistic GLMs for those individuals with tick infestation status in season 2 (respectively in season 3) as the response variable, the infestation status in season 1 (respectively season 2), species, sex and year as the explanatory variables, and the number of captures in season 2 (respectively season 3) as an offset term. As the infestation status of the previous season had no effect on the infestation status of the following season, we considered that these pseudo-replications would not bias the results of the model. 

We calculated the TBEV seroprevalence of small mammals per year, season, and species, and its exact 95% CI based on binomial distribution. The seropositive status of captured animals was modelled using a logistic GLM as a function of the small mammal species, season, and year. As few individuals were seropositive (see Results), we limited the number of variables included in the model. 

For all models, collinearity was checked in the models ensuring a variance inflation factor (VIF) <10 [72]. The backward elimination of explanatory variables was used to identify the most parsimonious model with the smallest Akaike information criteria (AIC). 

Statistical computations were performed in R 3.5.0 [73]. For all statistical tests, a *p*-value < 0.05 was considered statistically significant.

## Figures and Tables

**Table 1:** Proportion of small mammals infested by ticks or TBEV-seropositive, according to small mammal species, year, and season. The proportion of small mammals infested by ticks was calculated by dividing the individuals found to be infested by ticks (respectively TBEV-seropositive) for at least one capture session per season by the number inspected for ticks (respectively tested for TBEV antibodies). The proportion of small mammals being TBEV-seropositive was calculated by dividing the individuals found to be TBEV-seropositive for at least one capture session per season by the number tested for TBEV antibodies. Season 1: early April–early May, Season 2: early June–early July, Season 3: early September–early October.

Year	Season	No. Infested by Ticks/No. Inspected (%)			No. TBEV-Positive/No. Tested (%)		
		Tot	*Myodes*	*Apodemus*	Tot	*Myodes*	*Apodemus*
2012	Season 1	37/104 (35.6)	24/72 (33.3)	13/32 (40.6)	4/95 (4.2)	2/65 (3.1)	2/30 (6.7)
	Season 2	158/359 (44.0)	54/182 (29.7)	104/177 (58.8)	11/349 (3.2)	5/178 (2.8)	6/171 (3.5)
	Season 3	28/121 (23.1)	22/93 (23.7)	6/28 (21.4)	5/97 (5.2)	4/75 (5.3)	1/22 (4.5)
2013	Season 1	2/16 (12.5)	0/4 (0)	2/12 (16.7)	0/16 (0)	0/4 (0)	0/12 (0)
	Season 2	11/11 (100)	3/3 (100)	8/8 (100)	1/10 (10.0)	0/3 (0)	1/7 (14.3)
	Season 3	8/10 (80.0)	2/3 (66.7)	6/7 (85.7)	0/10 (0)	0/3 (0)	0/7 (0)
2014	Season 1	4/7 (57.1)	1/1 (100)	3/6 (50)	0/7 (0)	0/1 (0)	0/6 (0)
	Season 2	65/69 (94.2)	17/20 (85.0)	48/49 (98.0)	0/60 (0)	0/16 (0)	0/44 (0)
	Season 3	11/20 (55.0)	8/12 (66.7)	3/8 (37.5)	1/20 (5.0)	1/12 (8.3)	0/8 (0)

**Table 2:** Intensity of the infestation by larvae and nymphs per infested rodent-session in June–July (season 2) of 2012 and 2013.

	2012	2013
*No. of rodent-sessions in which ticks were collected*	67	12
Infested with nymphs/total infested rodent-sessions (%)	3/67 (4.5%)	6/12 (50.0%)
Infested with nymphs and larvae/total infested rodent-sessions (%)	2/67 (3.0%)	5/12 (41.7%)
*Tick infestation per rodent-session infested by ticks*		
Mean of larvae (+/− SD) per rodent-session infested by ticks	2.2 (2.2)	5.3 (4.8)
Median of larvae per rodent-session infested by ticks	2	4.5
Mean of nymphs (+/− SD) per rodent-session infested by ticks	0.1 (0.3)	0.6 (0.7)
Median of nymphs per rodent-session infested by ticks	0	0.5
*Larvae feeding on rodent-sessions infested by nymphs*		
Number of larvae (%) feeding on rodent-sessions infested by nymphs	4/147 (2.7%)	37/64 (57.8%)
Mean number of larvae per rodent-session infested by nymphs	2	7.4

## Supplementary Materials

The following are available online at https://www.mdpi.com/2076-0817/9/7/518/s1, Table S1: TBEV minimum infection rate (MIR) in questing *I. ricinus* nymphs and adults and density of infected nymphs (DIN) per 100 m² according to year and season. Confidence intervals of 95% (95% CI) were calculated using exact binomial law.

## References

[1] Bogovic P., Strle F. (2015). Tick-borne encephalitis: A review of epidemiology, clinical characteristics, and management. World J. Clin. Cases.

[2] Süss J. (2011). Tick-borne encephalitis 2010: Epidemiology, risk areas, and virus strains in Europe and Asia—An overview. Ticks Tick-Borne Dis..

[3] Mannelli A., Bertolotti L., Gern L., Gray J. (2012). Ecology of *Borrelia burgdorferi sensu lato* in Europe: Transmission dynamics in multi-host systems, influence of molecular processes and effects of climate change. FEMS Microbiol. Rev..

[4] Gray J.S., Kahl O., Lane R.S., Levin M.L., Tsao J.I. (2016). Diapause in ticks of the medically important *Ixodes ricinus* species complex. Ticks Tick-Borne Dis..

[5] Perret J.-L., Rais O., Gern L. (2004). Influence of climate on the proportion of *Ixodes ricinus* nymphs and adults questing in a tick population. J. Med. Entomol..

[6] Ernek E., Kozuch O., Lichard M., Nosek J., Albrecht P. (1963). Experimental infection of *Clethrionomys glareolus* and *Apodemus flavicollis* with tick-borne encephalitis virus. Acta Virol..

[7] Heigl Z., Von Zeipel G. (1966). Experimental infection with tick-borne encephalitis virus in *Clethrionomys glareolus*, *Apodemus flavicollis*, *Apodemus sylvaticus* and *Mus musculus*. 1. Virological studies. Acta Pathol. Microbiol. Scand..

[8] Kozuch O., Chunikhin S., Gresikova M., Nosek J., Kurenkov V., Lysý J. (1981). Experimental characteristics of viraemia caused by two strains of tick-borne encephalitis virus in small rodents. Acta Virol..

[9] Chunikhin S.P., Kurenkov V.B. (1979). Viraemia in *Clethrionomys glareolus* -a new ecological marker of tick-borne encephalitis virus. Acta Virol..

[10] Michelitsch A., Tews B.A., Klaus C., Bestehorn-Willmann M., Dobler G., Beer M., Wernike K. (2019). In Vivo Characterization of Tick-Borne Encephalitis Virus in Bank Voles (*Myodes glareolus*). Viruses.

[11] Randolph S.E. (2011). Transmission of tick-borne pathogens between co-feeding ticks: Milan Labuda’s enduring paradigm. Ticks Tick-Borne Dis..

[12] Hartemink N.A., Randolph S.E., Davis S.A., Heesterbeek J.A.P. (2008). The basic reproduction number for complex disease systems: Defining R0 for tick-borne infections. Am. Nat..

[13] Labuda M., Randolph S.E. (1999). Survival strategy of tick-borne encephalitis virus: Cellular basis and environmental determinants. Zentralblatt Für Bakteriol..

[14] Randolph S.E., Green R.M., Peacey M.F., Rogers D.J. (2000). Seasonal synchrony: The key to tick-borne encephalitis foci identified by satellite data. Parasitology.

[15] Randolph S.E. (1998). Ticks are not Insects: Consequences of contrasting vector biology for transmission potential. Parasitol. Today.

[16] Harrison A., Bennett N.C. (2012). The importance of the aggregation of ticks on small mammal hosts for the establishment and persistence of tick-borne pathogens: An investigation using the R0 model. Parasitology.

[17] Woolhouse M.E.J., Dye C., Etard J.-F., Smith T., Charlwood J.D., Garnett G.P., Hagan P., Hii J.L.K., Ndhlovu P.D., Quinnell R.J. (1997). Heterogeneities in the transmission of infectious agents: Implications for the design of control programs. Proc. Natl. Acad. Sci. USA.

[18] Burri C., Bastic V., Maeder G., Patalas E., Gern L. (2011). Microclimate and the zoonotic cycle of tick-borne encephalitis virus in Switzerland. J. Med. Entomol..

[19] Perkins S.E., Cattadori I.M., Tagliapietra V., Rizzoli A.P., Hudson P.J. (2003). Empirical evidence for key hosts in persistence of a tick-borne disease. Int. J. Parasitol..

[20] Randolph S.E., Miklisová D., Lysy J., Rogers D.J., Labuda M. (1999). Incidence from coincidence: Patterns of tick infestations on rodents facilitate transmission of tick-borne encephalitis virus. Parasitology.

[21] Rosà R., Pugliese A., Ghosh M., Perkins S.E., Rizzoli A. (2007). Temporal variation of *Ixodes ricinus* intensity on the rodent host *Apodemus flavicollis* in relation to local climate and host dynamics. Vector-Borne Zoonotic Dis..

[22] Perez G., Bastian S., Chastagner A., Agoulon A., Plantard O., Vourc’h G., Butet A. (2017). Ecological factors influencing small mammal infection by *Anaplasma phagocytophilum* and *Borrelia burgdorferi* s.l. in agricultural and forest landscapes: Tick-borne infection in small mammals. Environ. Microbiol..

[23] Kiffner C., Vor T., Hagedorn P., Niedrig M., Rühe F. (2011). Factors affecting patterns of tick parasitism on forest rodents in tick-borne encephalitis risk areas, Germany. Parasitol. Res..

[24] Rosà R., Tagliapietra V., Manica M., Arnoldi D., Hauffe H.C., Rossi C., Rosso F., Henttonen H., Rizzoli A. (2019). Changes in host densities and co-feeding pattern efficiently predict tick-borne encephalitis hazard in an endemic focus in northern Italy. Int. J. Parasitol..

[25] Perez-Eid C., Hannoun C., Rodhain F. (1992). The Alsatian tick-borne encephalitis focus: Presence of the virus among ticks and small mammals. Eur. J. Epidemiol..

[26] Zöldi V., Papp T., Rigó K., Farkas J., Egyed L. (2015). A 4-year study of a natural tick-borne encephalitis virus focus in Hungary, 2010–2013. EcoHealth.

[27] Crespin L., Verhagen R., Stenseth N.C., Yoccoz N.G., Prevot-Julliard A.-C., Lebreton J.-D. (2002). Survival in fluctuating bank vole populations: Seasonal and yearly variations. Oikos.

[28] Pucek Z., Jędrzejewski W., Jędrzejewska B., Pucek M. (1993). Rodent population dynamics in a primeval deciduous forest (Białowieża National Park) in relation to weather, seed crop, and predation. Acta Theriol. (Warsz.).

[29] Stenseth N.C., Viljugrein H., Jędrzejewski W., Mysterud A., Pucek Z. (2002). Population dynamics of *Clethrionomys glareolus* and *Apodemus flavicollis*: Seasonal components of density dependence and density independence. Acta Theriol. (Warsz.).

[30] Jensen T.S. (1982). Seed production and outbreaks of non-cyclic rodent populations in deciduous forests. Oecologia.

[31] Brugger K., Walter M., Chitimia-Dobler L., Dobler G., Rubel F. (2018). Forecasting next season’s *Ixodes ricinus* nymphal density: The example of southern Germany 2018. Exp. Appl. Acarol..

[32] Ostfeld R.S., Levi T., Keesing F., Oggenfuss K., Canham C.D. (2018). Tick-borne disease risk in a forest food web. Ecology.

[33] Krawczyk A.I., van Duijvendijk G.L.A., Swart A., Heylen D., Jaarsma R.I., Jacobs F.H.H., Fonville M., Sprong H., Takken W. (2020). Effect of rodent density on tick and tick-borne pathogen populations: Consequences for infectious disease risk. Parasit. Vectors.

[34] Dizij A., Kurtenbach K. (1995). *Clethrionomys glareolus*, but not *Apodemus flavicollis*, acquires resistance to *Ixodes ricinus*, the main European vector of *Borrelia burgdorferi*. Parasite Immunol..

[35] Kurtenbach K., Kampen H., Dizij A., Arndt S., Seitz H.M., Schaible U.E., Simon M.M. (1995). Infestation of rodents with larval *Ixodes ricinus* (Acari; Ixodidae) is an important factor in the transmission cycle of *Borrelia burgdorferi* s.l. in German woodlands. J. Med. Entomol..

[36] Pérez D., Kneubühler Y., Rais O., Gern L. (2012). Seasonality of *Ixodes ricinus* ticks on vegetation and on rodents and *Borrelia burgdorferi sensu lato* genospecies diversity in two Lyme borreliosis–endemic areas in Switzerland. Vector-Borne Zoonotic Dis..

[37] Hansmann Y., Pierre Gut J., Remy V., Martinot M., Allard Witz M., Christmann D. (2006). Tick-borne encephalitis in eastern France. Scand. J. Infect. Dis..

[38] Velay A., Argemi X., Wendling M.-J., Martinot M., Hansmann Y., Fafi-Kremer S. (2019). L’encéphalite à tique en France: qu’en savons-nous aujourd’hui ?. Rev. Francoph. Lab..

[39] Gaumann R., Muhlemann K., Strasser M., Beuret C.M. (2010). High-throughput procedure for tick surveys of tick-borne encephalitis virus and its application in a national surveillance study in Switzerland. Appl. Environ. Microbiol..

[40] Rieille N., Bressanelli S., Freire C.C.M., Arcioni S., Gern L., Péter O., Voordouw M.J. (2014). Prevalence and phylogenetic analysis of tick-borne encephalitis virus (TBEV) in field-collected ticks (*Ixodes ricinus*) in southern Switzerland. Parasit. Vectors.

[41] Carpi G., Bertolotti L., Rosati S., Rizzoli A. (2009). Prevalence and genetic variability of tick-borne encephalitis virus in host-seeking *Ixodes ricinus* in northern Italy. J. Gen. Virol..

[42] Radda A., Hofmann H., Kunz C. (1968). Experimentelle Infektion uniger heimischer Säugerarten mit dem Frühsommer-Meningo- Enzephalitis Virus. Zbl Bakt Mikr Hyg Abt Orig A.

[43] Nikitina N., Pchelkina A., Kovalevskaya Y.I. (1967). Study of persistence of antibody to tick-borne encephalitis virus in naturally occurring small rodents. Med. Parazit..

[44] Achazi K., Růžek D., Donoso-Mantke O., Schlegel M., Ali H.S., Wenk M., Schmidt-Chanasit J., Ohlmeyer L., Rühe F., Vor T. (2011). Rodents as sentinels for the prevalence of tick-borne encephalitis virus. Vector-Borne Zoonotic Dis..

[45] Labuda M., Kozuch O., Zuffová E., Elecková E., Hails R.S., Nuttall P.A. (1997). Tick-borne encephalitis virus transmission between ticks cofeeding on specific immune natural rodent hosts. Virology.

[46] Grindstaff J.L., Brodie E.D., Ketterson E.D. (2003). Immune function across generations: Integrating mechanism and evolutionary process in maternal antibody transmission. Proc. R. Soc. Lond. B Biol. Sci..

[47] Kallio E.R., Poikonen A., Vaheri A., Vapalahti O., Henttonen H., Koskela E., Mappes T. (2006). Maternal antibodies postpone hantavirus infection and enhance individual breeding success. Proc. R. Soc. B Biol. Sci..

[48] Gomez-Chamorro A., Heinrich V., Sarr A., Roethlisberger O., Genné D., Bregnard C., Jacquet M., Voordouw M.J. (2019). Maternal antibodies provide bank voles with strain-specific protection against infection by the Lyme disease pathogen. Appl. Environ. Microbiol..

[49] Cagnacci F., Bolzoni L., Rosà R., Carpi G., Hauffe H.C., Valent M., Tagliapietra V., Kazimirova M., Koci J., Stanko M. (2012). Effects of deer density on tick infestation of rodents and the hazard of tick-borne encephalitis. I: Empirical assessment. Int. J. Parasitol..

[50] Burri C., Korva M., Bastic V., Knap N., Avšič-Županc T., Gern L. (2012). Serological evidence of tick-borne encephalitis virus infection in rodents captured at four sites in Switzerland. J. Med. Entomol..

[51] Knap N., Korva M., Dolinšek V., Sekirnik M., Trilar T., Avšič-Županc T. (2012). Patterns of tick-borne encephalitis virus infection in rodents in Slovenia. Vector-Borne Zoonotic Dis..

[52] Tonteri E., Jääskeläinen A.E., Tikkakoski T., Voutilainen L., Niemimaa J., Henttonen H., Vaheri A., Vapalahti O. (2011). Tick-borne encephalitis virus in wild rodents in winter, Finland, 2008–2009. Emerg. Infect. Dis..

[53] Bakhvalova V.N., Potapova O.F., Panov V.V., Morozova O.V. (2009). Vertical transmission of tick-borne encephalitis virus between generations of adapted reservoir small rodents. Virus Res..

[54] Kozuch O., Gresíková M., Nosek J., Lichard M., Sekeyová M. (1967). The role of small rodents and hedgehogs in a natural focus of tick-borne encephalitis. Bull. World Health Organ..

[55] Couret J., Dyer M.C., Mather T.N., Han S., Tsao J.I., Lebrun R.A., Ginsberg H.S. (2017). Acquisition of *Borrelia burgdorferi* infection by larval *Ixodes scapularis* (Acari: Ixodidae) associated with engorgement measures. J. Med. Entomol..

[56] Randolph S.E., Gern L., Nuttall P.A. (1996). Co-feeding ticks: Epidemiological significance for tick-borne pathogen transmission. Parasitol. Today.

[57] Perez-Eid C. (1990). Les relations tiques—petits mammifères dans le foyer Alsacien d’encéphalite à tiques. Acarologia.

[58] Reye A.L., Hubschen J.M., Sausy A., Muller C.P. (2010). Prevalence and seasonality of tick-borne pathogens in questing *Ixodes ricinus* ticks from Luxembourg. Appl. Environ. Microbiol..

[59] May K., Jordan D., Fingerle V., Strube C. (2015). *Borrelia burgdorferi sensu lato* and co-infections with *Anaplasma phagocytophilum* and *Rickettsia* spp. in *Ixodes ricinus* in Hamburg, Germany: *Borrelia* infections in ticks in northern Germany. Med. Vet. Entomol..

[60] Labuda M., Nuttall P.A., Kozuch O., Elecková E., Williams T., Zuffová E., Sabó A. (1993). Non-viraemic transmission of tick-borne encephalitis virus: A mechanism for arbovirus survival in nature. Experientia.

[61] Mishaeva N.P., Erofeeva N.I. (1979). The effect of diapause of the tick *Ixodes ricinus* (*Ixodidae*) upon the multiplication of the tick encephalitis virus in them. Parazitologiya.

[62] Labuda M., Austyn J.M., Zuffova E., Kozuch O., Fuchsberger N., Lysy J., Nuttall P.A. (1996). Importance of localized skin infection in tick-borne encephalitis virus transmission. Virology.

[63] Zeipel G.V., Heigl Z. (1966). Experimental infection with tick-borne encephalitis virus in *Clethrionomys glareolus*, *Apodemus flavicollis*, *Apodemus sylvaticus* and *Mus musculus*. 2. Serological studies. Acta Pathol. Microbiol. Scand..

[64] Pérez-Eid C. (2007). Les tiques: Identification, biologie, importance médicale et vétérinaire..

[65] Quéré J.-P., le Louarn H. (2011). Les rongeurs de France: Faunistique et biologie.

[66] Beck C., Desprès P., Paulous S., Vanhomwegen J., Lowenski S., Nowotny N., Durand B., Garnier A., Blaise-Boisseau S., Guitton E. (2015). A high-performance multiplex immunoassay for serodiagnosis of flavivirus-associated neurological diseases in horses. BioMed Res. Int..

[67] Schwaiger M., Cassinotti P. (2003). Development of a quantitative real-time RT-PCR assay with internal control for the laboratory detection of tick borne encephalitis virus (TBEV) RNA. J. Clin. Virol..

[68] Puchhammer-Stöckl E., Kunz C., Mandl C.W., Heinz F.X. (1995). Identification of tick-borne encephalitis virus ribonucleic acid in tick suspensions and in clinical specimens by a reverse transcription-nested polymerase chain reaction assay. Clin. Diagn. Virol..

[69] Bestehorn M., Weigold S., Kern W.V., Chitimia-Dobler L., Mackenstedt U., Dobler G., Borde J.P. (2018). Phylogenetics of tick-borne encephalitis virus in endemic foci in the upper Rhine region in France and Germany. PLoS ONE.

[70] Cannon R.M. (2001). Sense and sensitivity—Designing surveys based on an imperfect test. Prev. Vet. Med..

[71] Schnabel Z.E. (1938). The estimation of total fish populations of a lake. Am. Math. Mon..

[72] James G., Written D., Hastie T., Tibshirani R. (2017). An Introduction to Statistical Learning.

[73] R Development Core Team (2018). R: A Language and Environment for Statistical Computing.

